# The RaDIANT community study protocol: community-based participatory research for reducing disparities in access to kidney transplantation

**DOI:** 10.1186/1471-2369-15-171

**Published:** 2014-10-28

**Authors:** Rachel E Patzer, Jennifer Gander, Leighann Sauls, M Ahinee Amamoo, Jenna Krisher, Laura L Mulloy, Eric Gibney, Teri Browne, Laura Plantinga, Stephen O Pastan, on behalf of the Southeastern Kidney Transplant Coalition

**Affiliations:** Department of Surgery, Division of Transplantation, Emory University School of Medicine, Atlanta, GA USA; Department of Epidemiology, Rollins School of Public Health, Emory University, Atlanta, GA USA; Emory Transplant Center, Atlanta, GA USA; Southeastern Kidney Council, Inc, Raleigh, NC USA; Department of Medicine, Section of Nephrology, Hypertension, and Transplant Medicine, Georgia Regents University, Augusta, GA USA; Piedmont Transplant Institute, Atlanta, GA USA; College of Social Work, University of South Carolina, Columbia, SC USA; Department of Medicine, Renal Division, Emory University School of Medicine, Atlanta, GA USA

**Keywords:** Kidney transplantation, Dialysis facility, Randomized trial, Education, Staff, Community-based participatory research

## Abstract

**Background:**

The Southeastern United States has the lowest kidney transplant rates in the nation, and racial disparities in kidney transplant access are concentrated in this region. The Southeastern Kidney Transplant Coalition (SEKTC) of Georgia, North Carolina, and South Carolina is an academic and community partnership that was formed with the mission to improve access to kidney transplantation and reduce disparities among African American (AA) end stage renal disease (ESRD) patients in the Southeastern United States.

**Methods/Design:**

We describe the community-based participatory research (CBPR) process utilized in planning the *R*educing *D*isparities *I*n *A*ccess to kid*N*ey *T*ransplantation (RaDIANT) Community Study, a trial developed by the SEKTC to reduce health disparities in access to kidney transplantation among AA ESRD patients in Georgia, the state with the lowest kidney transplant rates in the nation. The SEKTC Coalition conducted a needs assessment of the ESRD population in the Southeast and used results to develop a multicomponent, dialysis facility-randomized, quality improvement intervention to improve transplant access among dialysis facilities in GA. A total of 134 dialysis facilities are randomized to receive either: (1) standard of care or “usual” transplant education, or (2) the multicomponent intervention consisting of transplant education and engagement activities targeting dialysis facility leadership, staff, and patients within dialysis facilities. The primary outcome is change in facility-level referral for kidney transplantation from baseline to 12 months; the secondary outcome is reduction in racial disparity in transplant referral.

**Discussion:**

The RaDIANT Community Study aims to improve equity in access to kidney transplantation for ESRD patients in the Southeast.

**Trial registration:**

Clinicaltrials.gov number NCT02092727.

## Background

For the majority of the more than 600,000 patients in the United States (US) with end stage renal disease (ESRD) [1], kidney transplantation is the preferred treatment, providing longer survival, better quality of life, lower hospitalization rates, and substantial cost savings compared to dialysis [2, 3]. Despite these benefits, kidney transplantation is not available to all ESRD patients due to the paucity of available organs as well as long-standing racial disparities in access to both living donor (LD) and deceased donor (DD) transplantation [4–7]. Racial disparities in kidney transplant access are concentrated in the Southeastern US [4, 6, 8], where African American (AA) patients represent 67% of the prevalent ESRD population and where LD and DD transplant rates are the lowest in the nation [9, 10]. Targeting the ESRD regions with the most racial disparity in access to kidney transplantation could reduce the overall racial disparity in access to kidney transplantation [11]. However, despite numerous studies showing consistent racial disparities in access to transplantation, few interventions have been implemented to reduce disparities in the transplant process [12].

Our Southeastern Kidney Transplant Coalition (SEKTC) was formed in 2010 with the help of ESRD Network 6 (Southeastern Kidney Council) with a mission of improving equity in access to kidney transplantation for ESRD patients in the Southeast, particularly Georgia (GA), North Carolina (NC), and South Carolina (SC). This group consists of voluntary stakeholders in the ESRD community, including ESRD patients, dialysis facilities, transplant centers, social workers, organ procurement organizations, healthcare providers, academic researchers, patient advocacy groups within the tri-state region, and ESRD Network 6 staff.

At our inaugural SEKTC face-to-face meeting in February 2011, our SEKTC partners utilized a standard quality improvement approach to discuss root causes of delayed access to kidney transplantation in the Southeast to help inform the development of a multicomponent, quality improvement intervention [13]. A variety of patient-, provider-, dialysis facility-, neighborhood-, and health systems-level factors were identified as major barriers for the primarily AA ESRD patient population in the Southeast. These included: patient factors, such as lower income [14, 15], education [5], access to healthcare [16], education [5], medical comorbidities [17, 18], low health literacy [19], cultural beliefs and patient preferences [20–27], and limited social networks [28–30]; provider factors, such as misconceptions that AA patients do better on dialysis compared to transplant [23, 31–36], lack of education about kidney disease and the transplant process [37, 38], lack of information about the risks and benefits of treatment options among both patients and providers [31, 32], and provider bias [39–41]; and system-level factors, such as policies resulting in delays in access or outcomes on the basis of race, ethnicity, or socioeconomic status and non-standardized requirements for patient-provider discussions about transplantation [42].

The SEKTC members recognized significant gaps in our knowledge about the reasons for low kidney transplantation rates among AA ESRD patients in the Southeastern region. Our primary limitation was the lack of surveillance data on key steps in the transplantation process including lack of information on providers generating transplant referrals, provider perceptions on kidney transplantation, and patient-reported barriers to transplantation in the Southeast. Coalition members formed subgroup committees to address these gaps and, throughout the following 18 months, performed qualitative and quantitative research to improve our understanding of the challenges within the transplantation process in the Southeast. Detailed results from this population needs assessment [43–46] are described elsewhere, but we describe these results in brief below since these results influenced the development of multi-component intervention activities for the Reducing Disparities In Access to kidNey Transplantation (RaDIANT) Community Study.

Quantitative analyses of the Dialysis Facility Report data revealed significant variability in transplant rates across dialysis units in Network 6, where 80% of dialysis facilities were performing below the national average. Among the three states in ESRD Network 6, GA had the lowest kidney transplant rates, which were also the lowest rates in the entire nation [9]. Other quantitative analysis involved data from a dialysis-facility survey administered through our community partners. Results from this survey of more than 500 dialysis facility providers in GA, NC, and SC found that almost all providers (98.4%) reported they were comfortable discussing kidney transplantation with patients. However, most staff reported that >50% of their patients were not interested in transplantation [44]. Approximately 30% of providers believed that they did not have sufficient training about transplantation and did not have sufficient patient educational resources (33%) [44]. Qualitative research involved focus groups conducted among 40 ESRD patients from GA, NC, and SC. Most patients were interested in kidney transplantation, although many patients were confused about the kidney transplantation process. Common patient barriers derived from these focus groups included financial concerns, medical barriers, limited social network of transplant successes, and lack of information and education on transplantation within the dialysis facility [43].

The results of this population needs assessment helped our SEKTC partners to develop the multicomponent intervention activities. Specifically, the quantitative analyses that identified GA as the state with the lowest kidney transplant rates motivated our SEKTC to focus intervention activities within GA. In addition, the extreme variability in kidney transplantation across dialysis facilities suggested the need for traditional quality improvement methods for low performing dialysis facilities, including facility protocols to ensure best practices for kidney transplant referral. Dialysis facility surveys showed that targeted education among dialysis facility staff was needed, based on staff survey results showing that a third of providers felt they did not have sufficient patient educational resources and did not have sufficient training about transplantation. Focus group analyses suggested that patients had limited knowledge about the kidney transplant process and few educational resources or tools to help clarify misinformation or lacking information.

The results of this needs assessment, combined with practical aspects such as sustainability and feasibility, helped the SEKTC members develop a multicomponent, quality improvement intervention to deliver among dialysis facilities in order to reduce racial disparities in access to kidney transplantation. The purpose of this paper is to describe the community-based participatory research (CBPR) process used to design the protocol for the RaDIANT Community Study. The strategy and methods the SEKTC used to develop a large-scale, evidence-based, quality improvement intervention could serve as a model for other academic or community partnerships in developing and implementing interventions on the ESRD Network level.

## Methods

### Study overview

The RaDIANT Community Study is a dialysis facility-level, randomized clinical trial designed to test the effectiveness of a multicomponent intervention on improving patient referral for kidney transplantation within dialysis facilities. Prior to initiation of intervention activities, the RaDIANT Community Study was registered on clinicaltrials.gov (Protocol #NCT02092727). This study was approved by Institutional Review Boards at Emory Transplant Center, Georgia Regents Kidney and Pancreas Transplant Program, and Piedmont Transplant Institute for the collection of retrospective patient referral data, but no patient contact was made between researchers and patients.

### Target population, setting, and inclusion/exclusion criteria

Under the direction of Centers for Medicare & Medicaid Services (CMS), 18 regional ESRD Networks are responsible for the quality of care of patients with kidney disease in the US. ESRD Network 6 (GA, NC, and SC) has the largest ESRD patient population among the US ESRD Networks, servicing more than 600 dialysis facilities that treat ~40,000 patients. Approximately 47% of the dialysis facilities in ESRD Network 6 are located in GA, and these facilities treated more than 12,000 dialysis patients in 2012 [47]. The RaDIANT Community Study aims to target 134 outpatient dialysis facilities in GA, representing nearly half (47%) of all GA dialysis facilities.

**Figure 1:**
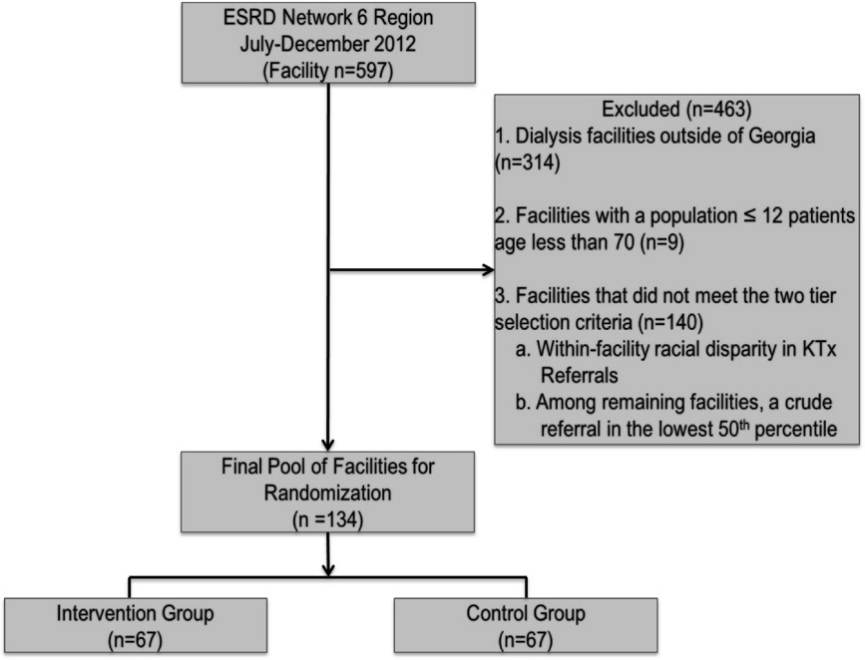
**RaDIANT community study flow diagram for dialysis facility selection.**

All dialysis facilities within GA were considered for randomization of the intervention (n = 283). To ensure that each facility had a large enough population to detect a change in the main effect of disparity reduction, facilities with a 2012 population of <25 patients (18-69 years of age) were excluded from the potential pool of facilities (n = 11). The remaining facilities were selected in a step-wise selection process: 1) The presence of a racial disparity in transplant referral (n = 75), or 2) crude annual referral in the lowest 50th percentile (referral <0.06; n = 59) for the state. The presence of a racial disparity was based on a difference of the proportion of AA ESRD patients referred and the proportion of white ESRD patients referred. The remaining facilities selected had a calculated crude referral in the lower 50th percentile. The final pool of 134 facilities were randomized by generating a random number to either the intervention (n = 67) or control (n = 67) group (Figure 1). Dialysis facilities in the intervention group are not blinded to intervention activities; however, facilities selected as controls are blinded. Researchers are not blinded to dialysis facility allocation.

Table 1 reports the characteristics of all dialysis facilities in Georgia (n = 283) and compares characteristics of the pool of dialysis facilities selected for potential randomization (*i.e*., meeting the two-tiered selection criteria) vs. facilities not selected for potential randomization. A greater proportion of patients within facilities selected for the RaDIANT Community study (vs. the facilities not selected) have higher proportions of AA (65.4% vs. 49.0%) patients compared to GA dialysis facilities not selected for the study. Dialysis facilities selected for the RaDIANT Community Study also have a higher proportion of patients with Medicaid insurance only (11.0% vs. 8.5%), patients who are unemployed (70.0% vs. 64.1%), and patients who have hypertension (91.4% vs. 88.1%) compared to facilities not selected for the study (p < 0.05 for each). As expected, facilities included in the RaDIANT Community Study have lower likelihood of placement on the national deceased donor waiting list and kidney transplant rates, compared to facilities that were not selected for randomization (Table 1).

**Table 1:** **Baseline characteristics of dialysis facilities, comparing all dialysis facilities in Georgia, the facilities selected for randomization, and dialysis facilities not selected for randomization**

Facility characteristic	Dialysis facilities in Georgia (n = 283)	Pool of dialysis facilities randomized for intervention (n = 134)	Remaining dialysis facilities in Georgia not selected for randomization (n = 149)	p-value
**Facility Demographic and Clinical Characteristics**				
# Patients per Facility, Mean, SD	46.0 ± 25.7	46.6 ± 25.5	45.4 ± 26.0	0.57
Number of Staff, Mean, SD	10.0 ± 7.4	10.1 ± 6.5	9.8 ± 8.1	0.48
For Profit, %	90.3	88.7	88.3	0.91
Average Age, Mean, SD	61.4 ± 5.9	61.2 ± 5.7	61.6 ± 6.1	0.76
% White	40.9	33.5	46.8	<0.001
% African American	56.5	65.4	49.0	<0.0001
% Uninsured	12.2	11.5	12.6	0.51
% Medicaid Only	9.1	11.0	8.5	0.01
% Unemployed	67.1	70.9	64.1	0.06
Time on Dialysis (Years), Mean, SD	4.9 ± 1.8	5.0 ± 1.21	4.8 ± 1.1	0.32
% Receiving No Access to Pre-ESRD Nephrology Care	27.3	26.0	28.3	0.28
% Not Informed of Transplant Options	2.8	3.1	2.4	0.28
% Diabetes	59.9	59.9	59.0	0.51
% Hypertensive	89.6	91.4	88.1	0.02
% AV Fistula	13.1	13.8	12.3	0.49
% of Incident Patients with AV Fistula	29.7	28.7	30.5	0.46
Average Count of Comorbidities, Mean, SD	3.0 ± 0.8	3.0 ± 0.8	2.9 ± 0.9	0.30
% ESA Prior to Dialysis	17.6	18.0	18.0	0.25
Standardized Mortality Ratio	1.06	1.07	1.05	0.41
**Transplant Access Measures at Baseline**				
% Waitlisted (Age < 70 yrs only)	17.8	15.5	20.4	<0.0001
Standardized Transplantation Ratio (2008–2011)	0.56	0.46	0.68	<0.001

### Study aims & objectives

The primary aim of the RaDIANT Community Study is to improve referral for kidney transplantation among AA ESRD patients in GA (primary outcome change in 1-year facility-level referral rate between intervention and control facilities). The secondary aim of the RaDIANT Community Study is to evaluate whether the implementation of this intervention reduced racial disparities in kidney transplant referral within and beyond the RaDIANT Community Study.

### Transplant referral data collection

The SEKTC recognized that a major gap in our knowledge of transplant disparities was the limited data available on patient- or facility-level referral for medical evaluation at a kidney transplant center, a key step in access to transplantation. Through our Coalition partners, we had the unique ability to access data on kidney transplant referrals from the transplant centers in the tri-state region. As this was the largest collection of transplant referral data collection to our knowledge, and because our data suggested that GA had the lowest kidney transplant rates, we chose to start tri-state data collection among the three adult transplant centers in GA: Emory Transplant Center (Atlanta, GA), Georgia Regents Kidney and Pancreas Transplant Program (Augusta, GA), and Piedmont Transplant Institute (Atlanta, GA). To ensure consistency of defining referrals, our Coalition partners – ESRD Network 6 – hosted several conference calls with CMS and other ESRD Networks that were collecting transplant referral data as part of the ESRD Network Statement of Work and Pilot Innovation projects. Partners agreed to define referral as when the transplant center received the patient’s referral form at the transplant center. The patient-level data collected from each transplant center included patient age, race/ethnicity, date of dialysis start, and date of transplant referral, evaluation, and waitlisting. Referral date was defined as the date in which the transplant center received a faxed transplant referral form from a dialysis facility or referring provider. Each transplant center securely sent patient-level referral data to ESRD Network 6, which served as the data coordinating center. ESRD Network 6 linked patient-level referral data with dialysis facility data by unique provider number using CROWNWeb. CROWNWeb is a web-based reporting system that allows dialysis facilities to report patient data. Following linkage with CROWNWeb, deidentified patient data were then linked to dialysis facility-level demographics using the publicly available 2012 Dialysis Facility Report data.

### Standard-of-care intervention (Study arm 1)

Dialysis facilities randomized to the standard-of-care intervention did not receive any specific quality improvement interventions related to transplant education. By law, all dialysis facilities are required to educate patients about transplantation as a treatment option and record the assessment of this in the patient’s Medicare eligibility (CMS-2728) form, although the quantity and quality of this education is unknown and may vary from facility to facility.

### Multicomponent intervention (Study arm 2)

Based on the results of the population needs assessment, the SEKTC aimed to develop a sustainable, multicomponent, multi-level quality improvement intervention to address disparities at the dialysis facility-, staff- and patient-level. These intervention activities were developed (or identified) by SEKTC members in face-to-face and phone conference meetings over several months. Final intervention activities were selected by the SEKTC Steering Committee members based on 1) feasibility, 2) sustainability, 3) core tenets of quality improvement interventions, and 4) perception of acceptance among stakeholders, including dialysis facilities, staff, and patients, as well as the larger kidney disease community.

The multicomponent, multi-level, quality improvement intervention targeted to dialysis facilities, staff, and patients includes educational webinars, staff- and patient-level educational activities, monthly monitoring of quality improvement activities, and traditional quality improvement oversight. Because ESRD Network 6 has significant experience with conducting large quality improvement interventions within dialysis facilities that may be performing worse than expected for a particular outcome, and because disparity reduction in transplant referral is part of the CMS Statement of Work for ESRD Networks [48], intervention activities are primarily delivered by ESRD Network 6. The ESRD Network retains the authority to mandate facility participation in quality improvement activities if deemed to have low performance; thus interventions are targeted to dialysis facilities that had either low overall kidney transplant referrals or those facilities that had a racial disparity within their facility (*i.e*., they had a lower proportion of AA vs. whites referred for kidney transplantation).

**Figure 2:**
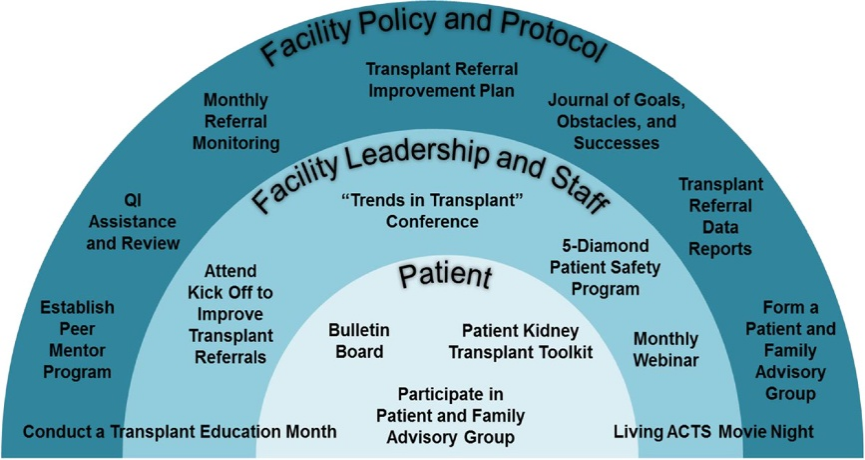
**Description of patient-, staff-, and facility-level interventions for RaDIANT community study.**

Intervention activities are designed to target and span multiple levels, including patient, facility staff, and facility protocol levels (Figure 2). For example, there are several interventions that target facility policies and protocols, including the requirement that facilities create their own transplant referral quality improvement plan by journaling their goals, obstacles, and successes; working with the ESRD Network on quality improvement assistance and review; creating a patient and family advisory group; conducting a transplant education month; hosting a movie night including culturally sensitive information about living donation; and establishing a peer mentoring program to help facilitate transplant recipients to speak to potential transplant candidates. In addition, facilities must report their monthly referrals to ESRD Network 6 and are given a baseline and mid-year report on their own facility’s performance related to transplant referral. Facility staff are recommended to attend their own facility’s kick off quality improvement session on improving transplant referrals, attend monthly educational webinars developed by SEKTC members, attend a transplant educational seminar or session across the state (including “Trends in Transplant” hosted by the Georgia Transplant Foundation, or Explore Transplant), and complete an online patient safety module. Educational activities targeting patients within facilities include bulletin boards that facility staff must set up to educate patients about transplantation, the opportunity to participate in the patient and family advisory group for transplantation, and the receipt of a patient educational “toolkit” about transplantation. The major intervention activities are described in more detail below.

### Educational webinars

ESRD Network 6 and various SEKTC members will host monthly webinars (45- to 55-minute internet educational seminars), which are intended to serve as a platform for all parties to discuss potential barriers to kidney transplantation, brainstorm ideas to overcome these obstacles, and celebrate successes. Webinars will cover the following topics: transplant quality improvement project overview, review of facility-level baseline transplant referral data feedback reports, how to establish a patient mentoring program, education materials for facility staff and patients, including Explore Transplant [49] and a shared patient/provider web- and mobile-based decision aid (iChoose Kidney; described below) that provides individualized mortality risks comparing dialysis vs. transplantation treatments that was co-developed by SEKTC partners, and a culturally sensitive DVD targeting the AA community [(Living About Choices in Transplantation and Sharing (ACTS) [50, 51]] detailing the advantages of LD kidney transplantation, financial issues related to transplantation, a question & answer discussion with transplant center outreach coordinators, a session on how staff can give patients an “elevator pitch” about kidney transplantation, a question & answer discussion with a transplant center medical director, and a “Patient’s Voice” session, during which transplant recipients talk about their personal experiences. Email reminders for webinar topics will be emailed to the dialysis facility project lead and project lead alternate staff member. Participation will be verified for each dialysis facility using a live check-in system; recorded webinars will be available following the live session.

### Patient and family educational programs

Implementation of patient and family education programs will be required at each dialysis facility in order to help inform individuals on the benefits of transplantation and information about how to navigate the transplant process. Along with dialysis facility-specific education programs, each facility will be provided with Living ACTS DVD and an electronic version of the Living ACTS booklet that could be printed and dispensed to patients and their family and help improve their knowledge of kidney transplant and the benefit of living donation. These materials are specifically designed for the AA community [51]. Dialysis facilities will also be encouraged to either develop a mentoring program that will connect patients and their families to former transplant patients, or utilize the Georgia Transplant Foundation’s (GTF) established program (the Dialysis Liaison Program) to match transplant recipients with dialysis patients within a facility [52]. SEKTC members developed a “peer mentoring toolkit” for facilities to provide recommendations for how mentors should be utilized within the facility to help improve access to kidney transplantation. A SEKTC partner, Georgia Transplant Foundation, will aid in tracking utilization of peer mentors at the facility level.

### Transplant referral data reports

**Figure 3:**
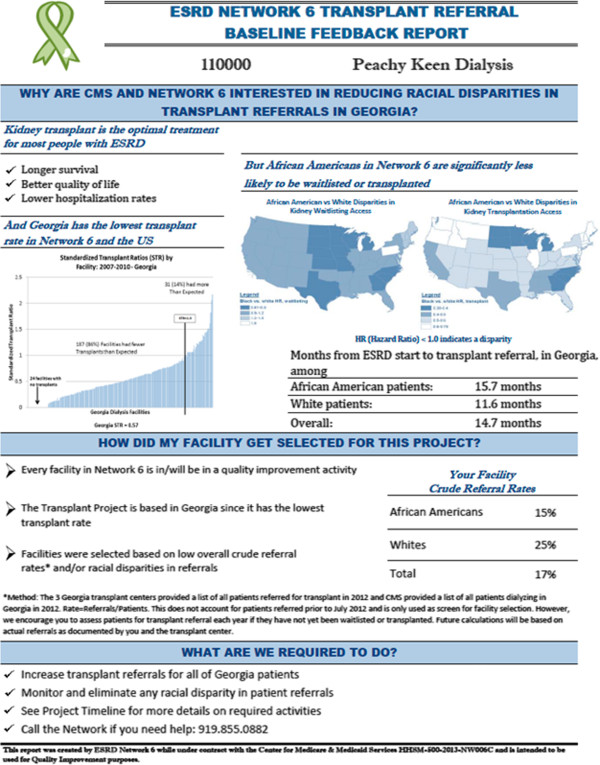
**Example kidney transplant referral feedback report for dialysis facilities.**

Similar to previous quality improvement interventions in ESRD Network 6 [47, 53–55], facilities will receive a baseline and mid-year facility-specific quality-of-care feedback report detailing their individual facility’s referral for kidney transplantation, including the overall proportion of patients referred as well as the proportion of white vs. AA patients referred, based on data reported from the three transplant centers in GA. The feedback reports also detail information about low kidney transplant rates and racial disparities in the average time from dialysis start to referral for ESRD patients in GA (Figure 3).

### Monthly monitoring of transplant referral data

Similar to previous dialysis facility quality improvement activities, ESRD Network 6 is responsible for monthly monitoring of the intervention facilities. Facilities will be required to submit a monthly report of the status of each patient in their facility, including whether the patient has been referred for kidney transplantation, and if so, whether they have been evaluated by the transplant center, waitlisted, or received a transplant. If the patient has not been referred, facilities must document the reason. At one year following the intervention, transplant centers will also report patient-level referral data to validate the monthly, facility-reported referral data and conduct the outcome evaluation.

### Quality improvement assistance and review

Dialysis facility-specific action plans to reduce disparities and to improve access to kidney transplant referrals will be a required component of this multicomponent intervention. Facility staff will receive detailed information (via email) about the core elements of quality improvement, including how to conduct a root cause analysis and identify a list of potential barriers. ESRD Network staff members will provide assistance and review of facility-specific action plans.

### Facility leadership and staff-level interventions

At baseline, dialysis facility staff members will be required to attend an informal orientation reiterating the importance of transplant. Dialysis facility leadership will be instructed by the ESRD Network staff members to host the orientation in order to provide details about the quality improvement project, but each facility has the flexibility for how and when they communicate the information to the facility staff. The orientation allows the facility to communicate with staff and show their dedication to improving referral rates, which can ultimately impact patient outcomes. To emphasize the commitment to improving transplant referrals, staff members will be encouraged to participate in Georgia Transplant Foundation’s symposium on “Trends in Transplant”—a free, educational conference that joins transplant physicians with potential candidates.

### Patient-level interventions

Facilities will be required to form Patient and Family Advisory Groups that are focused on increasing transplant referral. The Advisory Group is recommended to hold regular meetings to discuss referral activities, educational programs, and patient outreach. Along with the Advisory Group, patients will be given the opportunity to collaborate with dialysis facility staff in the creation of a “Kidney Transplant Toolkit”. This toolkit will give other dialysis facility patients a comprehensive overview of the transplant process including where to go for transplant evaluation, common questions and answers about the transplant process, potential barriers, and lessons learned from transplant recipients. Various educational resources (that have been previously developed and are easily accessible online) are included in this toolkit, including evidence-based patient education materials. The iChoose Kidney website and mobile application (iPhone and iPad tool) is an educational tool available to patients and their health care staff which uses patient age, sex, race, time on dialysis, and comorbidities to predict an individualized risk of mortality if the patient were to remain on dialysis vs. to receive a kidney transplant. The tool presents relative and absolute risks both numerically and graphically, utilizing best practices for health communication [56].

### Outcome measures

The primary outcome of the RaDIANT Community Study is the change in facility-level transplant referral (for patients <70 years) from baseline. Baseline referral will be calculated as the average number of unique referrals in a dialysis facility over the 12-month period preceding the intervention (January-December 2013) divided by the total number of ESRD patients within the facility for that time period. Post-intervention crude referral will be measured as the average number of unique referrals generated from January-December 2014 divided by the total number of ESRD patients within the facility for the same 12 month intervention period. As described above, referral will be defined as the receipt of a referral by the transplant center. Patient-level transplant referral data with facility identifiers will be collected from all of the transplant centers in GA and aggregated to summarize the total number of referrals to any transplant center in the state divided by the total number of adult ESRD patients <70 years in the facility. If facility characteristics are not equally distributed at baseline between control and intervention groups, we will consider adjustment using a Standardized Transplantation Referral Ratio (STRR). The STRR will be defined as the total number of observed transplant referrals within a facility divided by the total number of expected referrals based on the state average and adjusting for relevant covariates, among patients aged 18–69 [similar to the Standardized Transplantation Ratio (STR) used by CMS] [1].

A secondary outcome of interest is the reduction in AA vs. white racial disparity in transplant referral. The reduction in racial disparity will be measured as the difference in the proportion of referrals among AA vs. whites. Furthermore, we will examine whether the total number of AA ESRD patients referred for transplant in the state of GA increased from baseline to 12 months to examine the absolute impact of the RaDIANT Community Study on improving access for AA ESRD patients in the state.

### Other collected variables

#### Tracking of intervention activities

ESRD Network staff will monitor compliance with policies and procedures (*i.e*., forming a Patient and Family Advisory Committee for transplantation, participation in monthly webinars, submission of monthly patient-level facility-reported referral data, etc.) on an at least amonthly basis. Coalition partners, including Georgia Transplant Foundation, will monitor facility participation in the Peer Mentoring program by tracking requests for peer mentors and peer mentor activities within facilities. Georgia Transplant Foundation and transplant center partners will monitor facility staff participation in educational activities across the state, such as Trends in Transplant conferences and Explore Transplant events.

#### Patient and dialysis facility staff satisfaction with intervention activities

To examine feasibility of expanding the intervention outside of GA, the SEKTC members, with assistance from ESRD Network resources, will send an informal email survey (via survey monkey) to all facility project leadership in the study to evaluate the fidelity, reach, context, and sustainability of each intervention activity (target n = 134).

To examine the patient impact of the multicomponent interventions, a sample of patients within dialysis facilities will be surveyed on the relevance, knowledge, and readiness to pursue transplantation. Process evaluation data for both facilities and patients are described in Table 2.

**Table 2:** **Process evaluation data for medical directors and ESRD patients within dialysis facilities**

Dimension	Process evaluation question	Measurement
**Dialysis Facilities**		
Fidelity	To what extent was the intervention implemented as planned?	Checklist of each component of the intervention activity
Reach	To what extent did the intervention encourage participation?	Number of staff engaged; number of patients participating in intervention activities
Context	What were barriers and facilitators to implementing the intervention activities?	Open ended item at the end of the form
Sustainability	Are you willing to continue the intervention components at your facility indefinitely?	Yes/No Discrete Response for each intervention activity
**ESRD Patients**		
Participation	What was your level of participation in intervention activities?	Discrete level of participation item for each component of the intervention activity
Relevance	To what extent was the information offered relevant to you?	Checklist of each component of the intervention activity
Knowledge	Did the intervention component increase your knowledge about transplant options?	Self-reported assessment for each intervention component
Readiness	To what extent did the intervention component change your readiness to pursue transplant?	Measure of transplant readiness for each intervention component

### Statistical analyses

Descriptive analyses of facility-level baseline variables (demographic, clinical characteristics and transplant access measures) will be compared and differences between study arms at baseline will be evaluated using either *t* tests or their non-parametric equivalents for continuous variables and chi-square test for categorical variables. Significant differences in baseline characteristics will be adjusted when assessing the overall intervention effect as described above.

To evaluate the effect of the intervention on improvement in transplant referral by study arm, we will calculate the difference in facility-level transplant referral rate from baseline to 12 months for each facility in the study. Analysis of covariance (ANCOVA) will be used to test for post-intervention mean differences in referral rates for the control and intervention group after adjusting for the baseline referral rates and facility characteristics that differed significantly across the two arms at baseline. For skewed outcomes, differences in the intervention and control groups will be evaluated using a non-parametric Wilcoxon rank sum test.

For our secondary outcome, AA vs. white racial disparity in transplant referral, we will examine whether (i) the proportion of AA patients referred for transplantation increased from baseline to 12 month follow up at the facility level and (ii) whether there is a differential effect in the intervention group compared to the control group over time, after adjusting for between facility heterogeneity. A mixed effects model will be used to test for differences in the proportion of AA patients referred between baseline and 12-month follow up in the two study arms respectively. Fixed main effects of (a): time (measured in months from baseline; baseline assumed month 0) (b): intervention status (control = 0, intervention = 1), and (c) their interaction on referral disparity, averaged across different subpopulation of facilities will be assessed. Facility level random effects (intercepts and slopes) will be considered to adjust for variations in the average time trend due to unmeasured confounding independent of the intervention. If the individual facility trajectories indicate a similar time trend (slope), a random intercept only model will be sufficient to account for between facility heterogeneity at baseline.

To examine the absolute increase of the intervention on AA ESRD patient referrals, patient-level logistic regression analysis will be performed with intervention status, race (AA versus whites) as primary independent variables and controlling for patient-level baseline characteristics. Because patients are clustered within facilities, we will adjust for the clustering effect. Interactions between intervention and race will be included to test for the differential intervention effects by race. For interpretation of findings, we will compare and contrast the standardized predicted referral rates by racial group within the intervention and control facilities, respectively.

### Power and sample size calculations

Our sample size calculations are based upon our primary aim to improve access to kidney transplant referral and also feasibility and resource availability for intervention activities. To test the primary null hypothesis of no difference in referral proportions between the control and intervention groups, our sample size of 134 dialysis facilities (67 facilities in each arm), with an estimated 46 patients per facility, achieves 80% power to detect a moderate difference of 3.1% to 4.8% in referral proportions between the two groups at 5% significance level. The power calculation assumes a 14% crude referral proportion in the control group based on baseline referral data and an intra-cluster correlation coefficient (ICC) of 0.01 to 0.05, respectively, to account for between-facility variation.

## Discussion

In this paper, we described the formation of an academic–community partnership; the development of a multicomponent, quality improvement intervention based on results of a community needs assessment; and the selection of dialysis facilities for the RaDIANT Community Study. Few interventions have been conducted to reduce disparities in the early steps of the transplant process [12] and, to our knowledge, no study has described the development of a large-scale, dialysis facility-level, randomized study to improve transplant referral on a state-wide level and to reduce disparities in access to transplantation. This is the first study that we are aware of that collected transplant referral data from collaborating transplant centers on a state level. The detailed description of the development of a multicomponent intervention could help other ESRD Networks, transplant centers, dialysis facilities, or members of the kidney community to develop similar strategies to improve access to transplantation. In addition, other researchers or community members may find that the unique academic, community, and government partnership we utilized in this study protocol may be a feasible way to utilize sustainable interventions within the community to reduce health disparities.

Reasons for racial disparities are multifactorial and may include patient, provider, and system factors [8]. Nearly one-third of ESRD patients are not informed of transplant as a treatment option at the time of ESRD diagnosis [57], and while AA patients comprise a disproportionate part of the ESRD population, AA patients are less likely to be informed about transplantation than white patients [31, 32]. This may be particularly important for our primarily AA ESRD population in the Southeast, where kidney transplant rates are the lowest in the nation [9]. Systemic, coordinated quality improvement initiatives targeting dialysis facilities, including arteriovenous fistula access and influenza vaccination rates [53], have previously resulted in improved access to high-quality care for ESRD patients, and similar interventions may hold promise in reducing disparities in access to kidney transplantation.

Community and academic partnerships can be a useful way to deliver population-based interventions and may have a high potential for reducing health disparities. Targeting ESRD regions in the Southeast, the region with the most racial disparity in transplant access, holds promise for reducing the overall racial disparity in access to kidney transplantation [11]. The RaDIANT Community Study aims to reach more than one-third of the AA ESRD patients in GA within nearly half of dialysis facilities in the state with the lowest kidney transplant rate in the nation. The results of the RaDIANT Community study could help reduce disparities in access to kidney transplantation for AA ESRD patients living in the Southeastern US.

## Abbreviations

AA: African-American
ANCOVA: Analysis of covariance
CBPR: Community-based participatory research
CMS: Centers for Medicare & Medicaid Services
DD: Deceased donor
ESRD: End-stage renal disease
GA: Georgia
GTF: Georgia Transplant Foundation
LD: Living donor
NC: North Carolina
RaDIANT: Reducing Disparities In Access to kidNey Transplantation
SC: South Carolina
SEKTC: Southeastern Kidney Transplant Coalition
STRR: Standardized transplantation referral ratio.
